# Three-Month Magnetic Resonance Imaging Post-Prostate Artery Embolization Predicts 1-Year Clinical Outcomes

**DOI:** 10.1007/s00270-025-04254-w

**Published:** 2025-11-03

**Authors:** Conrad von Stempel, Lazaros Tzelves, Sukhmani Khangura, Yakup Kilic, Miles Walkden, Ahmed Al-Nowfal, Graham Munneke, John Hines, Giorgio Mazzon, Zafer Tandoğdu

**Affiliations:** 1 Department of Interventional Radiology, UCLH, London, UK; 2 Division of Surgery and Interventional Science, UCL, London, UK; 3 Department of Urology, UCLH, Westmorland Street, London, UK

**Keywords:** Predictors, Clinical success, Infarction

## Abstract

**Purpose:**

To investigate whether post-prostate artery embolization (PAE) magnetic resonance imaging (MRI) can predict clinical outcomes at 1 year.

**Materials and Methods:**

Retrospective study of 63 consecutive patients undergoing microsphere PAE for benign prostatic enlargement. Clinical assessments were performed with the International Prostate Symptom Score (IPSS), quality of life (QoL) scores, and non-invasive urodynamics. MRI was performed at baseline and 3-month post-PAE: intravesical protrusion, prostate volume (PV), and infarction volumes were measured with semi-automated volumetry. Clinical success was defined as (1) a reduction of IPSS to < 9 and QoL score to < 3 or (2) freedom from urinary catheter in catheter-dependent patients. Statistical modeling was performed with multivariate logistic regression.

**Results:**

Sixty-three patients were included. Median PV 116 ml (IQR 85–153); median PV reduction 26% (IQR 19–45) at 3 months. Presence of infarction on 3-month MRI was associated with clinical success at 12 months (OR = 1.15, 95% CI: 1.04–1.36, *p* = 0.025). A receiver operator curve analysis of infarct volume returned an area under the curve of 0.77 (CI 0.66–0.89) and indicated that an infarct volume ≥ 5% had a sensitivity of 72% and specificity of 80% to predict clinical success. PV reduction did not show statistically significant correlation with clinical success (*p* = 0.348, 95% CI: 0.98–1.08). A lower baseline IPSS was correlated with better clinical outcomes (OR 0.78; 95% CI 0.64–0.91; *p* = 0.003). Other clinical and anatomical variables including intravesical protrusion were not correlated with clinical outcomes.

**Conclusion:**

Lower baseline IPSS and presence of infarction of > 5% on 3-month post-PAE MRI were associated with better clinical outcomes at 12 months.

**Graphical Abstract:**

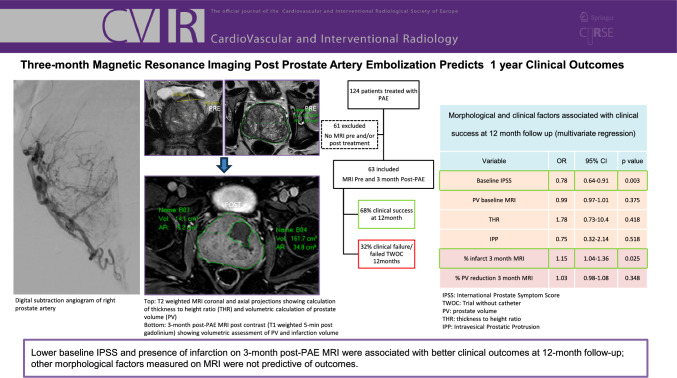

## Introduction

Prostate artery embolization (PAE) is a minimally invasive treatment for bladder outflow obstruction due to benign prostatic enlargement (BPE) [1–3]. Although effective, up to 20% of patients experience clinical failure in the first 2 years post treatment [4]. There is no widely accepted imaging biomarker to assess response to PAE or predict intermediate or long-term clinical benefit.

Post-PAE international prostate symptom scores (IPSS) have been shown to improve by up to 20 points by one to three months, with IPSS reduction plateauing after this [5] and longer clinical success rates reported at 87% at 12 months and over 75% at five years [4–6]. Prior studies have identified several potential predictors of PAE outcome, including patient age [7], embolization technique [6, 8] baseline prostate, and median lobe morphology [9–11]. The changes in the gland post-PAE on magnetic resonance imaging (MRI) are well documented in the literature, with the description of transition zone infarcts described on post-contrast MRI [12] and the presence of greater transition zone volume reduction (but not infarction) correlating with better clinical outcomes [13]. Greater clinical success was correlated with the presence of infarction on MRI performed at 3-month post-PAE in one study [14] and an increased prostate-specific antigen blood level at 24-h post-PAE as a surrogate marker of ischemia post-PAE [7].

Identification and quantification of infarction may provide an imaging biomarker to help predict longer-term clinical outcomes and inform early discussions with patients about repeat or alternative therapies. This study investigates whether biometrics measured on MRI performed at 3-month post-PAE are correlated with clinical success at 12 months.

## Methods

A retrospective cohort study of all patients treated with PAE for BPE between January 2019 and January 2023 with at least 12-month follow-up was performed. Inclusion and exclusion criteria are detailed in Table 1.

**Table 1:** Inclusion and exclusion criteria

Inclusion criteria:
Bilateral prostate artery embolization
Multiparametric MRI prostate performed pre-PAE (within 12-months of procedure) and at 3-months post-PAE
Both catheter-dependent and non-catheter-dependent patients were included
Exclusion criteria:
Lack of MRI at baseline and/or follow-up
Lack of ≥ 12-month follow-up

MRI – magnetic resonance imaging

PAE – prostate artery embolization

### Embolization Technique

PAE was performed after informed written consent and under local anesthetic with conscious sedation in selected cases. Access was either through the common femoral artery (5 French sheath; 5 French reverse curve catheter, Pisco, Merit medical®; 2.4 French microcatheter, Progreat, Terumo®) or the left radial (5 French Slender sheath, Terumo®; 5 French Berenstein 150 cm, Merit medical®; 1.9 French, 175 cm microcatheter, (Truselect, Boston®) loaded with a 0.014 inch guidewire (Fathom, Boston®). Embolization was performed with calibrated 250 micron and 400 micron hydrogel microspheres (Embozene, Varian®) following the PErFecTED technique [15]. Shunt embolization was performed with 2 mm and 3 mm coils (Hilal, Cook®), and coil back of the prostate artery was performed in all cases with 3 mm and 4 mm coils (microNestor, Cook®). An indwelling catheter was placed in patients with prostates measuring over 150 ml.

### Imaging Follow-up

**Fig. 1:**
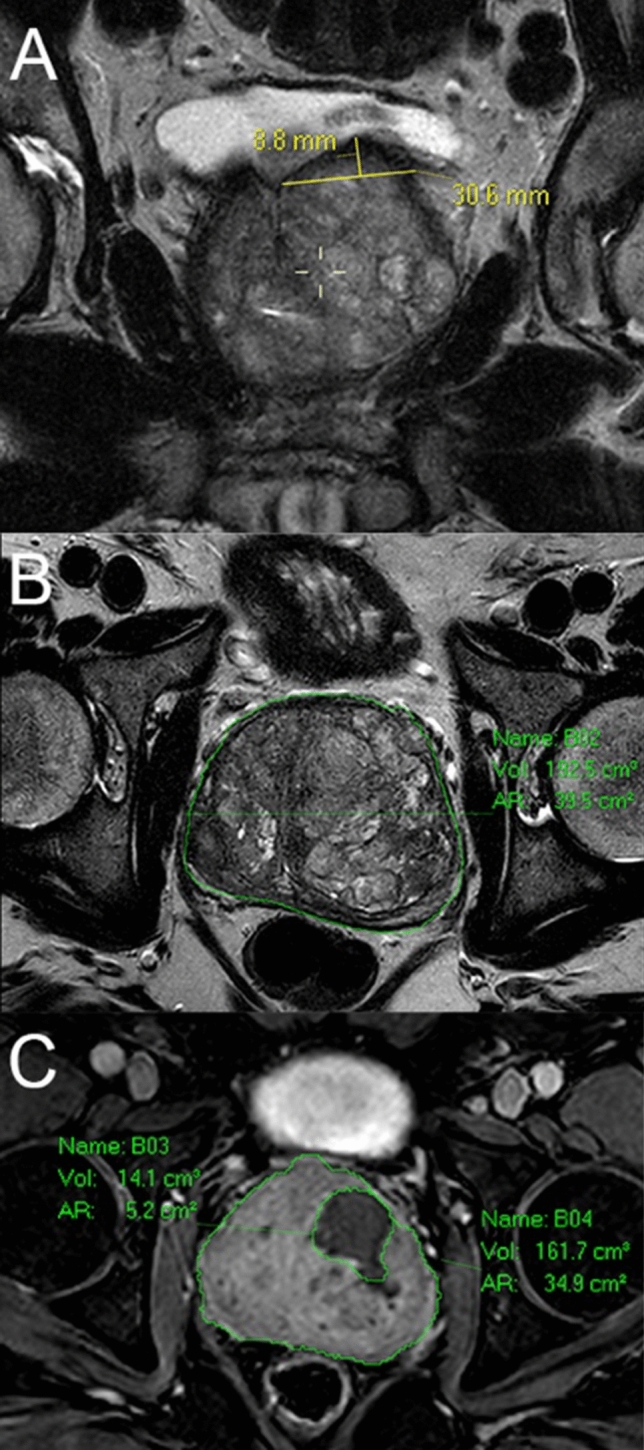
MRI Pre and Post-PAE **A** – Coronal T2 image of the prostate with the thickness (30.6 mm) and height (8.8 mm) of the intravesical prostatic protrusion measured. The calculated THR would be 3.4 **B** – Axial T2 image of the prostate with semi-automated volumetry of the gland shown (192.5 ml) **C** – T1 fat-saturated post-contrast image at 5 min delay showing the total post-PAE PV on the right-hand side (161.7 ml) [constituting a volume reduction by 16%] and the volume of the infarcted portion (14.1 ml) shown on the left-hand side. This constitutes an infarction percentage of 8.6%. MRI – magnetic resonance imaging, PAE – prostate artery embolization, T1 – Time 1 weighted, T2 – Time 2 weighted, THR – thickness-to-height ratio

Multiparametric MRI of the prostate was performed at baseline and at 3-month post-PAE as per local standard of care protocol, using axial and coronal Time-2 weighted (T2) sequences, diffusion weighted image (DWI), and dynamic contrast-enhanced (DCE) fat saturated T1-weighted sequences. Prostate volume (PV) was calculated with a semi-automated volumetry software (Livewire segmentation, Carestream, Philips®) using T2 axial images. The intravesical prostatic protrusion (IPP) and thickness-to-height ratio (THR) of the median lobe using the technique described by Yu et al. (see Fig. 1) [11] were measured.

Post-PAE infarction was defined as non-enhancing regions on 5 min post-contrast images. Infarct volume was measured using Livewire segmentation, summing both lobes to provide a single value, which was expressed as a percentage of the whole post-PAE PV (see Fig. 1c). All MRI scans were independently reviewed on two separate occasions by a consultant interventional radiologist (C.v.S) and a senior radiology fellow (Y.K.) blinded to the clinical outcome measurements, and the mean of the two values obtained was used for analysis.

### Clinical Follow-up

Clinical assessment was performed at day 1, 7, and at 3, 6, and 12-month post-PAE. Reported symptoms of post-embolization syndrome, including fever, dysuria, and pain, as well as ulceration on the penis and bleeding from the rectum, were recorded. In addition to MRI, patients underwent non-invasive urodynamics at 3 and 12 months, with measurement of peak flow rate and post-void residual. For catheter-dependent patients, a trial without catheter (TWOC) was performed 2-week post-PAE and scheduled for a repeat trial 2 weeks later. If patients had repeated failure, they were deemed unsuccessful PAE and offered training on intermittent self-catheterization and referred for consideration of transurethral resection of the prostate. Successful TWOC was defined as remaining catheter-free up until the end of the study or at least 12 months. At 3 and 12 months, IPSS questionnaires were completed and QOL scores were assessed.

### Outcome Measures

The primary outcome measure was clinical success at 12-month follow-up, defined as a reduction in IPSS to < 9, and a QoL score of < 3 and/or a successful trial without catheter (TWOC) in previously catheter-dependent patients, defined as remaining catheter-free for at least 12-month post-PAE.

Secondary outcomes were percentage reduction in total PV on MRI at 3-month post-PAE and volume and percentage of infarction of the prostate on post-contrast MRI, quantified as the non-enhancing region relative to total post-PAE PV.

### Statistical Tests

All statistical analyses were performed using both SPSS (Version 30.0.0, IBM) and R statistical software (Version 4.5.0, Austria, Vienna), independently by two authors (C.V.S. and L.T.). Descriptive statistics were calculated, including medians, interquartile ranges (IQR), and variance. Q-Q plots and the Shapiro-Wilk test were used to test for normal distribution of data. Univariate analysis was performed with the Mann-Whitney U test and Chi-squared test. Univariate analyses were used to compare demographic, MRI-derived, and clinical variables between clinical success and failure groups (see appendix 1 for details).

Multivariate analyses were performed using variables with univariate significance and known clinical relevance from the literature.

Two multivariate binary logistic regression models were tested:

- **Model 1 (Pre-procedural only):** Included baseline clinical and anatomical variables (age, BMI, LTC, IPSS, THR, and PV).
- **Model 2 (Combined):** Included all variables from Model 1 plus 3-month post-PAE MRI-derived features: percentage infarction and percentage PV reduction at 3 months.

Receiver operator curve characteristics (ROC) were constructed for the statistically significant predictors identified in multivariate analysis, and optimal values for sensitivity and specificity were calculated. Significance was set at *p* < 0.05.

## Results

**Fig. 2:**
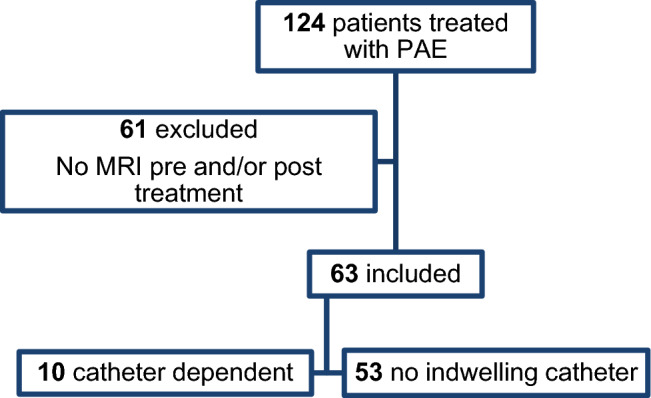
Flow diagram showing the included patient cohort. PAE – prostate artery embolization

One hundred and twenty-four patients underwent PAE during the study period; 63 met the inclusion criteria, with a median age of 72 years (IQR 68–72) and a median PV of 116 ml (IQR 87–154). Ten patients (16%) were catheter-dependent at baseline. The median total procedure time was 110 min (IQR 93–135), with a median fluoroscopy time of 40 min (IQR 27–50.5) and a median radiation dose area product of 288 gy/cm^2^ (IQR 182–408) (see Fig. 2).

Fifteen cases (23%) were performed from a single common femoral artery approach, 48 (77%) from a left radial approach. Two prostatic arteries (PA) were identified in 54% of cases, 39% had three or more, while 7% had only one identifiable PA supplying the gland. The most common PA origins were type 1 (36%) and type 4 (29%) according to classifications by de Assis (Table 2) [16]. Prostatic arterial shunts to the rectum, penis, or bladder were identified in 26 cases (41%), and in 7 cases (11%) protective coils were placed in these to reduce the risk of non-target embolization, while in the remainder subselective catheterization or competing flow was used to prevent non-target embolization.

**Table 2:** Origins of PA and additional PA according to De Assis 16 classification

Origin of PA type	Left PA	Additional left PA	Right PA	Additional right PA	Total
I [with superior vesical]	15	7	17	4	43 (36%)
II [after superior vesical]	6	0	7	0	13 (11%)
III [ from obturator]	6	3	5	2	16 (14%)
IV [from internal pudendal]	13	2	15	4	34 (29%)
V [ other origin]	6	1	5	0	12 (10%)

Prostate artery (PA)

### Complications

**Fig. 3:**
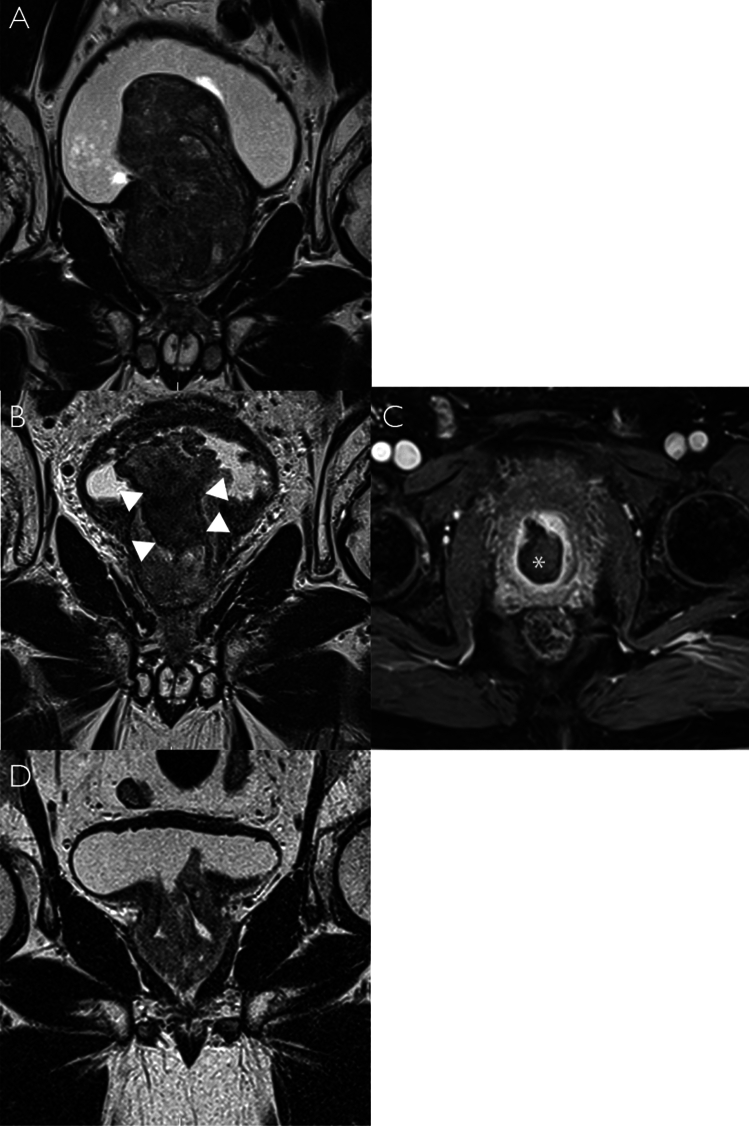
MRI prior to PAE, at 3 months and 12 months post-PAE **A** Coronal T2 image showing a 165 ml prostate gland with a large median lobe **B** Coronal T2 image showing the infarcted median lobe within a cavity in the prostate (white arrows) and **C** Axial post-contrast T1 image showing the non-enhancing sloughed tissue (white asterisk) **D** Coronal T2 image at 12 months showing significant PV reduction and interval removal of the sloughed median lobe

Three patients developed acute urinary retention within 3 months post-PAE. Two required cystoscopy removal of infarcted tissue (grade 3 complication as per Cirse Quality Assurance Document and Standards for Classification of Complications [17]); one spontaneously passed the sloughed median lobe per urethra. In all three cases, the 3-month MRI identified the sloughed tissue within the bladder (see Fig. 3).

Mild post-embolization syndrome was reported by 82% of patients, including dysuria (in those without catheters) and pain for a median of 10-day post-PAE.

### MRI Follow-up

**Fig. 4:**
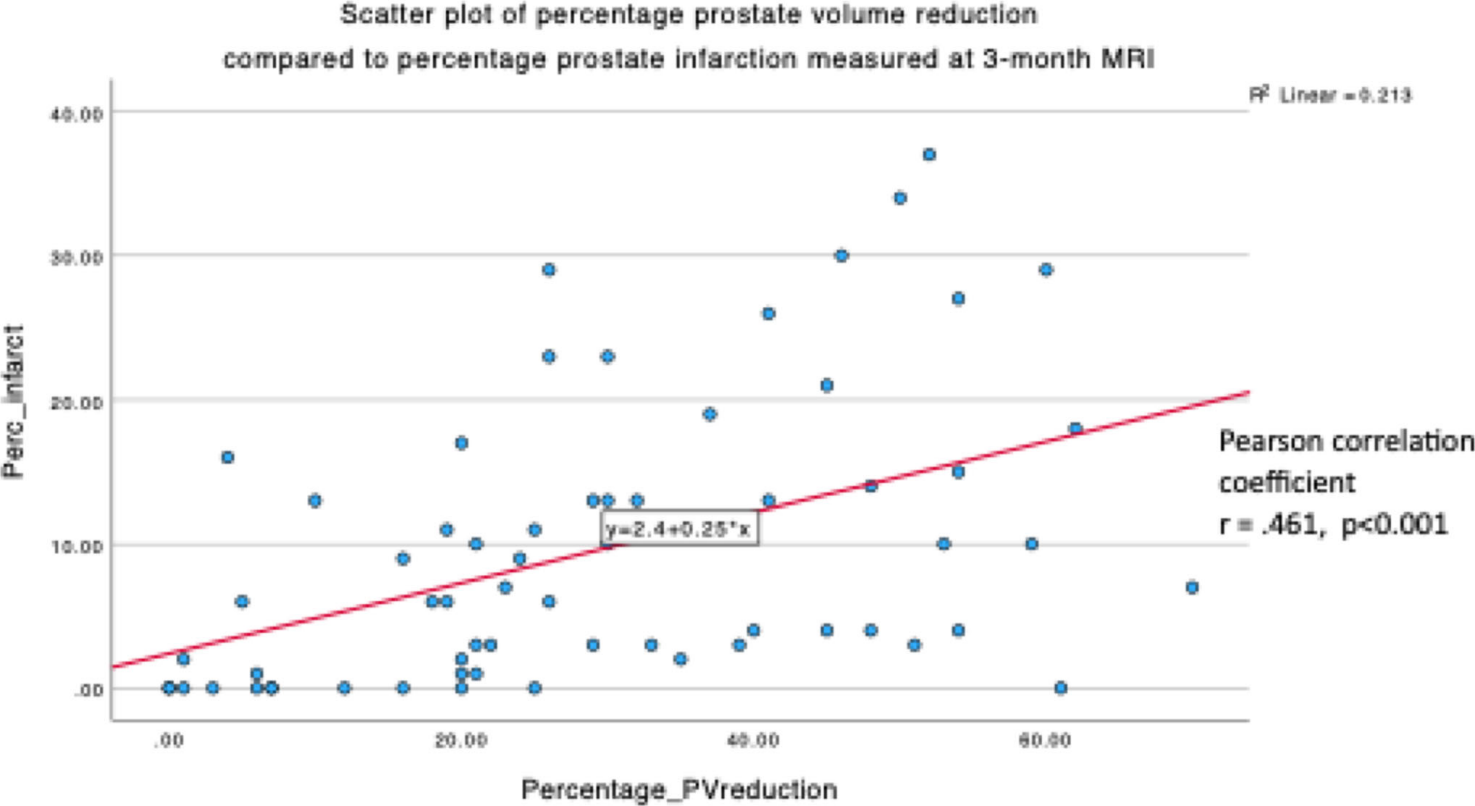
Scatter plot with line of best fit drawn to show the relationship between percentage PV reduction and percentage prostate infarction on the 3-month post-PAE MRI

At 3-month post-PAE, MRI measured median PV was 74 ml (IQR 85–153), constituting a median reduction of 26% (IQR 18–45). The transition zone demonstrated a greater reduction in volume compared to the peripheral zone (20% vs. 3%), and infarction was identified in 81% of patients with a median volume of 6 ml (IQR 2–14) and percentage of post-PAE PV of 7% (IQR 2–13). Pearson product-moment correlation testing was performed to determine the relationship between percentage PV reduction and percentage prostate infarction: there was a statistically significant positive correlation (*r* = 0.461, *n* = 63, *p* < 0.001) (see Fig. 4).

### Clinical Follow-up

Median clinical follow-up was 15.5 months (IQR 13–24) and clinical success was achieved in 43 (68%). Univariate tests showed that successful outcomes were associated with: a lower baseline IPSS score (median IPSS 19 vs 25 in failure; *p* = 0.003); greater post-PAE percentage PV reduction (median PV reduction: 30% (IQR 21–48) versus 18% (IQR 4.5–36.5); *p* = 0.01) and a greater percentage of prostate infarction, 10% (4–18) versus 1.5% (0–4); *p* < 0.001 (see Table 3).

**Table 3:** Difference in measured variable according to treatment outcome and odds ratio of positive outcomes (univariate analysis)

Variable	Overall	Success	Fail	*p*-value	OR	95% CI	*p*-value
Sample size	63	43 (68.3%)	20 (31.7%)				
Age (years)	72 (65.5–78)	71 (64.5–71)	74 (67.5–80)	0.24	0.96	0.90–1.03	0.260
BMI (kg/m^2^)	26 (25–28.5)	26 (25–29)	25.5 (25–28)	0.61	1.09	0.94–1.30	0.268
PV (ml)	115 (85.5–152)	116 (90–153.5)	93 (73.5–142)	0.19	1.01	0.99–1.02	0.196
Baseline IPSS	22 (18–26)	19 (16–23)	25 (22–30)	0.003	0.85	0.75–0.94	0.006
Baseline Qmax	6.8 (4.9–8.9)	7 (5.2–8.9)	6.2 (4.5–8.7)	0.35	1.08	0.93–1.30	0.348
Baseline PVR	129 (52–212)	113 (50–212)	130 (96–204)	0.63	1.00	0.99–1.01	0.979
LTC	10 (15.9%)	7 (16.3%)	3 (15%)	0.99	1.11	0.27–5.59	0.880
THR	1.1 (0.82–1.66)	1.13 (0.83–1.60)	0.97 (0.75–1.92)	0.57	1.36	0.78–3.16	0.379
IPP	1.80 (1.30–2.4)	1.80 (1.30–2.20)	1.85 (1.25–2.61)	0.97	1.04	0.71–1.65	0.835
Number PA	2 (2–3)	2 (2–3)	2 (2–3.5)	0.23	0.63	0.32–1.16	0.144
% infarct	7 (2–13)	10 (4–17)	1.5 (0–4)	< 0.001	1.12	1.04–1.24	0.011
% PV reduction	26 (19–45)	30 (21–47)	18 (5–35)	0.01	1.04	1.01–1.08	0.018

IPSS: international prostate symptom score, BMI: body mass index, PV: Prostate volume; IPSS: International Prostate Symptom Score; Qmax: Peak flow rate; PVR: post-void residual, LTC: long-term catheter, THR: thickness-to-height ratio of middle lobe, IPP: intravesical protrusion; PAs: prostatic arteries

### Multivariate Models

Model 1 (pre-procedural parameters only): the model was statistically significant, χ2(4) = 16.2, p 0.013 and correctly classified 78.8% of cases. Among predictors, only baseline IPSS was significantly associated with PAE success. However, ROC analysis showed that baseline IPSS as an independent variable was a poor outcome predictor (AUC = 0.25, 95% CI: 0.10-0.39).

Model 2 incorporated both pre-procedural variables and 3-month post-PAE MRI findings (percentage infarction and PV reduction at 3 months). The model was statistically significant, *χ*^2^(4) = 17.5, *p* < 0.025 and correctly classified 84% of cases. Infarction percentage was an independent predictor of clinical success (OR 1.15, 95% CI: 1.04–1.36, *p* = 0.025), while PV reduction was not (OR 1.03, 95% CI: 0.98–1.08, *p* = 0.348). Baseline IPSS remained a significant predictor (OR 0.78, 95% CI: 0.64–0.91, *p* = 0.003). ROC AUC was 0.77 (CI 0.66–0.89) and an optimal value of 5% infarction had a sensitivity of 72% and specificity of 80%. Percentage PV reduction was not associated with clinical success (*p* = 0.348, 95% CI: 0.98–1.08) (see Table 4).

**Table 4:** Multivariate Logistic Regression of Pre- and Post-PAE Predictors of Clinical Success

Variable	Odds ratio	95% CI	p-value
Multivariate analysis 1 Pre-procedural variable			
PV	0.999	0.985–1.014	0.922
THR	1.843	0.632 – 5.374	0.263
Baseline IPSS	0.826	0.722–0.944	0.005
Age	0.937	0.857–1.025	0.158
Presence of LTC	131	0.000–13	0.999
BMI	1.072	0.853–1.346	0.551
Multivariate analysis 2: Combination of Pre-procedural and 3-month MRI variables			
Baseline IPSS	0.78	0.64–0.91	0.003
PV	0.99	0.97–1.01	0.375
THR	1.78	0.73–10.4	0.418
Height of middle lobe	0.75	0.32–2.14	0.518
% infarct	1.15	1.04–1.36	0.025
% PV reduction	1.03	0.98–1.08	0.348

IPSS: international prostate symptom score, BMI: body mass index, PV: Prostate volume; IPSS: International Prostate Symptom Score; Qmax: Peak flow rate; PVR: post-void residual, LTC: long-term catheter, THR: thickness-to-height ratio of middle lobe, IPP: intravesical protrusion; PAs: prostatic arteries

## Discussion

A key contribution of this study is that the identification and confirmation of 3-month post-PAE infarction percentage on MRI is an independent predictor of long-term clinical benefit, determined as a reduction of IPSS to below 9 points and successful removal of urinary catheter. Specifically, an infarction volume of greater than five percent at 3 months was predictive of clinical success at 12 months, with a sensitivity and specificity of 72% and 89%, respectively. These findings suggest that 3-month post-PAE prostate MRI may be a potential imaging biomarker for longer-term success of PAE.

This was demonstrated through the development of two logistic regression models: a pre-procedural model using only baseline anatomical and clinical variables, and a second model that included 3-month post-PAE MRI findings. The addition of 3-month MRI data improved model performance, increasing the explained variance from 37 to 56% and improving classification accuracy from 78.8 to 84%. In this combined model, infarction percentage remained a statistically significant predictor, whereas PV reduction did not. A greater baseline IPSS value showed a positive correlation with increased clinical failure at 12 months (OR 0.78, 95% CI: 0.64–0.91, *p* = 0.003); this is a recognized predictor of failure of medical therapy for BPE as well as a potential predictor for clinical failure of PAE [7, 10, 18]. Other clinical factors, including the presence of long-term catheter and imaging-derived variables such as intravesical protrusion, the thickness-to-height ratio of the median lobe, and the number of prostatic arteries on invasive angiography were not associated with 1-year clinical outcomes.

PAE achieved symptomatic relief through ischemia-induced infarction, which reduces both PV and prostatic smooth muscle tone [19, 20]. This has been corroborated with ultrasound elastography studies post-embolization [21]. Several clinical studies have described infarction on MRI prostate, between 1 and 3 months post-PAE, and the rise in PSA post-PAE, as a surrogate marker of infarction, predicting clinical success [7, 14]; although results have been inconsistent [12, 13]. Overall gland reduction reaches its peak after 3 months, and PV reduction is correlated with clinical success [5, 22]; yet even in patients with significant reduction in PV of more than 15%, there may be minimal clinical benefit [23]. Although the presence of infarction is associated with a more significant reduction in PV, it can occur without significant total PV reduction [5, 15].

These data show that both prostate percentage infarction and PV reduction were significantly greater in patients with clinical success on univariate testing; however, on multivariate models, the significance of total gland volume reduction was lost. This suggests that the dominant mechanism of successful PAE is infarction of the prostate. Despite the observed significant positive correlation between infarction and PV reduction, the transition zone may demonstrate significant infarction without necessarily corresponding overall PV reduction. Different embolization techniques, embolic size, and agent choices have been suggested to promote prostate infarction by improving the obliteration of the entire intraprostatic arterial collateral network that might otherwise lead to reperfusion and early clinical failure [15, 24, 25]; yet, there is no accepted imaging biomarker to predict clinical outcomes. Infarcts within the prostate are readily identified on MRI [12] and some groups have suggested a correlation between infarction and improved clinical outcomes [14]. This study provides a quantification of the changes in the prostate on MRI at 3 months and correlates these with 12-month outcomes; however, it is limited due to the retrospective study design and intermediate follow-up time, as well as the heterogeneous group of patients, including both those with and without long-term catheter dependency. Furthermore, clinical success was largely determined by patient-reported scores or catheter-free rates, rather than objective measurements.

## Conclusions

Infarction volume on 3-month MRI is associated with clinical success at 12 months and may serve as a marker of treatment durability following PAE. A threshold of ≥ 5% infarction at 3 months may serve as a practical imaging biomarker for guiding patient management. Prospective validation is required to confirm its utility in guiding patient management.
